# Efficiency of the Austrian disease management program for diabetes mellitus type 2: a historic cohort study based on health insurance provider’s routine data

**DOI:** 10.1186/1471-2458-12-490

**Published:** 2012-06-29

**Authors:** Herwig Ostermann, Victoria Hoess, Michael Mueller

**Affiliations:** 1 Division for Health Policy, Administration and Law, Department of Public Health and HTA, University for Health Sciences, Medical Informatics and Technology, Eduard Wallnöfer-Zentrum I, 6060, Hall in Tyrol, Austria; 2 Department for Health Economics, Austrian Health Institute, Stubenring 6, 1010, Vienna, Austria; 3 Unit for Health Management, Social Insurance Institution for Business, Wiedner Hauptstraße 84-86, 1050, Vienna, Austria

## Abstract

**Background:**

The Austrian diabetes disease management program (DMP) was introduced in 2007 in order to improve health care delivery for diabetics via the promotion of treatment according to guidelines. Considering the current low participation rates in the DMP and the question of further promotion of the program, it is of particular interest for health insurance providers in Austria to assess whether enrollment in the DMP leads to differences in the pattern of the provision of in- and outpatient services, as well as to the subsequent costs in order to determine overall program efficiency.

**Methods:**

Historic cohort study comparing average annual levels of in- and outpatient health services utilization and its associated costs for patients enrolled and not enrolled in the DMP before (2006) and 2 years after (2009) the implementation of the program in Austria. Data on the use of services and data on costs were extracted from the records of the Austrian Social Insurance Institution for Business. 12,199 persons were identified as diabetes patients treated with anti-diabetic medication or anti-diabetics with insulin throughout the study period. 314 diabetics were enrolled in the DMP.

**Results:**

Patients enrolled in the diabetes DMP received a more evolved pattern of outpatient care, featuring higher numbers of services provided by general practitioners and specialists (79 vs. 62), more diagnostic services (22 vs. 15) as well as more services provided by outpatient care centers (9 vs. 6) in line with increased levels of participation in medical assessments as recommended by the treatment guideline in 2009. Hospitalization was lower for DMP patients spending 3.75 days in hospital, as compared to 6.03 days for diabetes patients in regular treatment. Overall, increases in costs of care and medication throughout the study period were lower for enrolled patients (€ 718 vs. € 1.684), resulting in overall costs of € 5,393 p.c. for DMP patients and € 6,416 p.c. for the control group in 2009.

**Conclusions:**

Seen from a health insurance provider’s perspective, the assessment of the Austrian diabetes DMP shows promising results indicating improved quality of outpatient care as well as overall cost advantages due to the lower hospitalization rates. Due to methodological limitations of the retrospective study and to the restricted data access, further promotion of the DMP must be accompanied by prospective research and preferably controlled trials in order to provide a solid basis for the decision of whether to include diabetes DMP into the insurer’s basic benefit package.

## Background

Diabetes is commonly regarded as one of the major causes for premature deaths worldwide. According to estimates of the world diabetes foundation, 285 million people were affected by diabetes worldwide in 2010 with an expected rise of up to 438 million people by 2030 and an overall increase in prevalence from 6.4% (2010) to 7.8% (2030). Moreover, substantial regional variances can be observed with highly industrialized countries such as the United States (8+) or, to a lesser extent, European Union Member States (3-6%, Austria 4%) featuring the highest share of diabetics because of particular lifestyles prevalent in these countries associated with the acquisition of diabetes mellitus type 2. Consequently, diabetes poses a substantial burden on health care systems not only due to the fact of lifelong treatment and the subsequent more frequent use of health care services, but also due to severe long-term sequelae as well as complications associated with unmanaged diabetes, such as adult blindness, amputations of extremities, cardiovascular diseases.

As the treatment of (severe) diabetes usually involves lifelong therapy with anti-diabetic medication and/or the injection of insulin as well as regular monitoring of the patients’ health status and subsequent patient involvement, diabetes disease management programs (DMPs) have been developed in most health systems of industrialized countries. Building on the standardization of treatment pathway as well as the implementation of treatment guidelines, DMPs are intended to lead to coordinated or even integrated health care delivery and ultimately better health outcomes for participating individuals at reasonable additional or even lower costs [1-3].

However, empirical evidence appears to be quite heterogeneous in terms of the outcomes of diabetes DMPs [4]: Whereas several studies found evidence of improved disease control in terms of intermediate measures such as HbA1C measurements or eye examinations [5-10], several authors raised methodological concerns as to whether these effects are caused by the mere nature of the DMP or specific biases, mostly selection biases [10-13]. For health insurance providers, empirical evidence for changes in utilization of health services and financial outcomes is inconclusive. Several research groups [6,10,14,15] have identified reductions in health service utilization and overall costs, whereas other authors reported evidence for increased utilization and costs [11] or inconsistent evidence for a reduction in service utilization [12,16] or costs [17].

Based on the patient records of diabetics covered by one of the mandatory health insurance providers in Austria, the authors therefore seek to assess whether participation in the disease management program for type 2 diabetics would result in different patterns of outpatient health care service utilization and hospital admission rates as well as different numbers of prescribed medication. Taking into account the costs of outpatient health services received as well as cost estimates for inpatient care the authors also aim to assess whether the economic rationale of disease management can be met by the Austrian diabetes DMP.

## Methods

### Population

The authors were granted permission to analyze the insurer’s records of diabetes patients covered by the Social Insurance Institution for Business in Austria, which is part of the Austrian mandatory health insurance system and covers self-employed and retired self-employed persons as well as their spouses and children (unless insured by another social insurance institution due to occupation). In the year 2009 there were 705,607 persons insured by the Social Insurance Institution for Business, 21,299 of whom could be identified as diabetes patients treated with either oral medication and/or insulin. Diagnosed diabetes patients only following diabetic diets could not be identified, as health care providers are not obliged to report data on diagnosis to Austrian social insurance institutions [18].

Out of the subtotal of 21,299 patients treated with either anti-diabetic medication and/or insulin in 2009, 14,408 were identified as diabetics that had already been treated in 2006. Due to the fact that enrollment onto the Austrian diabetes DMP was not possible before the beginning of 2007, only this subgroup of 14,408 patients was included in the subsequent analysis as figures for 2006 would reflect baseline values in order to assess the impact of participation. Moreover, in order to identify type 1 diabetics, who do not represent the target population of the Austrian DMP and also differ from type 2 diabetics in terms of disease control, the authors performed a separate analysis according to medication intake due to the lack of outpatient diagnosis data in general as well as non-existent access to hospital discharge data. Having excluded patients receiving insulin only, 12,199 diabetics treated with either oral anti-diabetics or oral anti-diabetics with insulin could finally be identified as the study population, out of whom 314 persons were enrolled in the diabetes DMP by 2009.

Table 1 summarizes the characteristics of enrolled as well as non-participating type 2 diabetics with regard to sex and age. Whereas no statistically significant difference could be identified in terms of gender distribution, diabetics enrolled in the program were on average 3.9 years younger than their non-participating counterparts. Further socio-demographic data such as income, employment status or education were not provided by the Social Insurance Institution for Business because of data protection issues and the subsequent risk of violating personal rights. However, due to the specific target population of the insurance institution mainly representing self-employed or retired self-employed persons as well as their spouses and children respectively, overall statistics show that the collective of those insured is quite homogenous in terms of their socio-demographic characteristics. The specific population also accounts for the high share of male diabetes patients, which is also reflected by a higher portion of men who are insured in general (61.4% vs. 38.6%) [19].

**Table 1:** Demographic characteristics of diabetes patients

		**2009**		
		**Enrolled patients (N = 314)**	**Non-participating patients (N = 11,885)**	**P Value**
Age - yr. (mean +/− SD)		66.5 +/− 9.9	69.4 +/− 11.3	<0.001
Sex - no. (%)				0.170
	Male	219 (69.7)	7,848 (66.0)	
	Female	95 (30.3)	4,037 (34.0)	

Sex was compared with Pearson's chi-square test, otherwise the Mann–Whitney *U*-test was used.

### Characteristics of the Austrian diabetes DMP

In 2004 the Federation of the Austrian Social Insurance Institutions mandated the Social Insurance Institution of Styria to lead and coordinate the development of a diabetes DMP. The “Therapie Aktiv” program was presented in 2006 with the first diabetes patients enrolled in the program in 2007.

As for other disease management programs the Austrian diabetes DMP is geared towards type 2 diabetics (even though a share of approx. 10% of enrolled diabetics was treated with insulin only and had to be excluded from the study population) and builds upon patient education, promotion of self-management, patient registry and evidence based treatment pathways incorporating international research as well as recommendations of the Austrian Society for Diabetes [20]. Overall the program aims at (1) the achievement of optimal blood sugar management, (2) the avoidance or delay of associated long-term sequelae and complications, (3) the reduction of risk of strokes and heart attacks, (4) the avoidance of harmful side effects of therapy as well as (5) the promotion of active and self-responsible patient cooperation [21].

Participation in the Austrian diabetes DMP is voluntary for both patients as well as physicians, who may either be general practitioners or internists in private practice. Whereas there is no financial incentive for patients to enroll in the program, participating physicians benefit from a lump sum payment of € 100 p.a. for each enrolled patient. Moreover, physicians receive extra reimbursement for primary consultation to the amount of € 55 as well as for the delivery of additional therapeutic medical services inherent to the DMP.

Participating physicians are obliged to do compulsory training in the field of diabetes management in advance as well as continuing education. The program features compulsory checklists as well as a standardized reporting system in order support treatment according to guidelines and coordination of care of all physicians involved in the process [20]. More enhanced models of cooperation amongst different providers of outpatient and inpatient care (’integrated care’) along with adequate payment mechanisms have not been devised so far, as provision of outpatient care in Austria is predominantly based on self-employed individual physicians, who generate the majority of their income on a fee-for-service basis [22].

### Outcome measures

As pointed out above, the full set of diabetes patients’ records for outpatient care for 2009, the selected parameters on hospitalization (cases, length of stay) and the overall cost and utilization data for 2006 were made available to the authors in order to analyze the effects of the Austrian diabetes DMP in terms of health services utilization and process quality of care as well as its economic effects (cost-cost analysis). In particular, the following outcome measures were assessed by the authors:

Outpatient service utilizationOutpatient service utilization was derived by calculating the average number of reimbursed outpatient services per patient provided by each health profession in 2009. Reimbursed services include outpatient services provided by general practitioners and specialists as well as outpatient care centers and other health professionals. Moreover, services provided by physicians not having a contractual relationship with the Social Insurance Institution for Business (’private physicians’) are also included in the insurer’s records as patients receive reimbursement for four fifths of the contracted fee by the insurance institution after having consulted a private physician.

HospitalizationHospital treatment as well as length of stay was generated on hospital admission and discharge data included in the insurer’s patient records. In order to provide homogenous and comparable figures on hospitalization rates, the authors included only inpatient cases in terms of overnight stays. Figures for diabetes patients treated in hospitals’ outpatient clinics could not be collected because only the outpatient health services within the regular hospital setting (i.e. 0-days stay) could be identified in the admission and discharge data. The majority of regular ambulatory cases treated in hospitals’ outpatient clinics are not reported to the insurer, because services provided in outpatient clinics of hospitals are funded by the centrally pooled hospital funds on the basis of lump-sum payments [22,23].Moreover, hospitalization rates only reflect inpatient treatment in public hospitals. Treatment in private hospitals not covered by the Social Insurance for Business is not included. Because public hospitals account for more than 90% of all hospital stays in Austria and as private hospitals mainly reflect small hospitals (sanatoria) focusing on selective treatments, hospitalization for acute care is mainly represented by admissions to public hospitals.

Treatment according to guidelinesIn order to analyze process quality of diabetes care the authors assessed the frequency of specific services delivered to patients as recommended by the reference guideline [21]: (1) glycosylated hemoglobin (HbA1C) testing, (2) retinal testing, (3) performed electrocardiogram, (4) serum lipid testing, (5) microalbumin assessment, and (6) examination of the status of blood vessels.

Costs of care and medicationThe average annual costs of outpatient care and medication for diabetes patients for 2006 (i.e. before the start of the DMP) and 2009 were derived by adding the costs of all reimbursed outpatient services (see also outpatient service utilization) to the costs of prescribed medication.Due to the central funding of the Austrian DRG-system (’Leistungsorientierte Krankenanstaltenfinanzierung’), which features a retrospective reimbursement of hospitals administrated by the hospital funds (and not by each social insurance institution individually), primary data on costs of inpatient treatment are not included in the insurer’s patient records [23]. In order to derive overall costs, the authors included cost calculations for inpatient care by multiplying average days for inpatient care with average costs for hospital care as published by the Austrian ministry of health [24].

### Data analysis

The study represents a retrospective cohort study comparing average annual levels of in- and outpatient health services utilization as well as its associated costs for patients enrolled and not-enrolled in the DMP for diabetes mellitus type 2. Statistical testing was performed using PASW Statistics 18, after having adjusted the group of non-participating diabetes patients for age and sex. The authors used Pearson’s chi-square test for binary variables (sex, patients hospitalized) and the Mann–Whitney *U*-test for ordinal metrics (treatment according to guidelines). For continuous variables (frequency of in- and outpatient services utilization, costs) the Kolmogorov-Smirnov D-Test was applied in order to test for normal distribution. As all continuous variables tested did not meet the criterion of normal distribution, the authors used the Mann–Whitney *U*-test in order to identify statistically significant differences. Statistical tests ware applied two-sided with the confidence interval set at 95%.

## Results

### Outpatient service utilization

Table 2 summarizes the average number of outpatient services received by type 2 diabetics for both study groups in 2009. Overall, patients enrolled in the DMP received an average number of approx. 185 reimbursed services p.a., whereas their counterparts receiving regular diabetes treatment received approx. 191 reimbursed services.

**Table 2:** Health services utilization

	**Average number of outpatient services received (2009)**			
Profession*	**Enrolled patients(N = 314)**	**Non-participating patients(N = 11,885)**	**Difference**	**P-Value**
Medical services				
Internal medicine	15,89	9,08	6,81	<0.001
General medicine	48,90	41,66	7,24	<0.001
Ophtalmology	6,01	3,66	2,35	<0.001
Dentistry	3,88	3,32	0,56	0.061
Urology	2,50	2,29	0,21	<0.001
Orthopedics	2,16	2,25	−0,09	<0.001
Diagnostic services				
Laboratory service	13,11	8,97	4,14	<0.001
Computer/Magnetic Resonance Tomography	3,16	1,57	1,59	<0.001
Radiology	5,38	4,27	1,11	0.978
Outpatient care centers				
Ambulatory health care centers	6,41	4,14	2,27	<0.001
Outpatient clinic for physical medicine	2,79	1,67	1,12	0.158
Other health care practitioners				
Physiotherapy	2,10	1,78	0,32	0.105
Non-medical services				
Orthopedic shoemaking	0,32	0,15	0,17	<0.001
Hearing aid acoustician	0,07	0,05	0,02	0.109
Wound care bandaging	17,23	31,24	−14,01	0.903
Transport service	7,61	31,09	−23,48	0.019
Other	17,62	19,55	−1,93	<0.001
All services**	185,21	190,65	−5,44	<0.001

* Professions cumulatively accounting for 90% of total reimbursement for outpatient services are included only.

** All services include all reimbursed outpatient services.

All variables were compared with the Mann–Whitney *U*-test.

Table 2 shows major differences between the types of use of outpatient health services: Patients participating in the DMP received a higher number of services provided by general practitioners and medical specialists in private practice (79 vs. 62), as well as diagnostic services (22 vs. 15) and services provided by outpatient care centers (9 vs. 6). On the other hand, non-medical services such as wound care bandaging (17 vs. 31) and transport services (8 vs. 31) were more frequently provided to patients not enrolled in the DMP.

When only looking at professions with significant differences in terms of services provided to both groups (p < 0.05), the composition of the identified professions again reflects the different pattern of health services utilization. Even more explicitly it can be shown that medical professions usually involved in diabetes treatment (general practitioners, internists and ophthalmologists) as well as ambulatory health care centers provide a significantly higher number of services to DMP patients and also more laboratory services. For non-medical services the utilization of orthopedic shoemaking and transport services turned out to differ significantly: Patient participating in the DMP received orthopedic shoemaking more often whereas transport services were provided more frequently to the control group.

### Hospitalization

The differences within hospitalization rates of type 2 diabetics either enrolled in the DMP or receiving regular treatment are presented in Table 3. In the overall share of patients being hospitalized in 2009, 35.0% of enrolled patients received inpatient treatment whereas 35.6% of non-participating patients were sent to hospital. When considering the average number of hospital stays as well as the lengths of stay, the differences between the study groups become more distinct: the hospitalization of DMP patients accounted for 1.79 stays and 10.71 hospital days in 2009 whereas his/her counterpart receiving regular treatment had 2.24 stays and 16.94 hospital days, with the latter parameter (days spent in hospital) representing a significant difference (p < 0.05). Overall, diabetics enrolled in the DMP spent 3.75 days in hospital in 2009 as compared to 6.03 days in hospital for the control group, though this difference does not turn out to be significant for a 95% confidence interval.

**Table 3:** Hospitalization rates of diabetes patients

	**2009**		
	**Enrolled patients (N = 314)**	**Non-participating patients (N = 11,885)**	**P Value**
Patients hospitalized - no. (%)	110 (35.0)	4,233 (35.6)	0,833
Average number of hospital stays (all patients)	0.63	0.80	0.636
Average days spent in hospital (all patients)	3.75	6.03	0.381
Average number of hospital stays (patients hospitalized)	1,79	2,24	0.259
Average days spent in hospital (patients hospitalized)	10,71	16,94	0.007
Average length of hospital stays (patients hospitalized)	6,32	7,82	0.019

The share of patients hospitalized was compared with Pearson's chi-square test, otherwise the Mann–Whitney *U*-test was used.

### Treatment according to guidelines

Table 4 provides an overview of various indicators of process quality of diabetes care by summarizing the frequency of specific medical assessments performed for both study groups as recommended by the reference guideline for diabetes treatment [21]. Overall, the analyzed data show that patients enrolled in the DMP are more likely to receive structured diabetes care according to guidelines. All assessed medical services inherent to evidence based disease management were provided more frequently to enrolled patients(p < 0.001). In 2009 HbA1C measurements and serum lipids testing accounted for the most substantial differences in the share of patients having received no assessment in 2009 (12.7% vs. 36.6% for HbA1C, 14.3% vs. 38.1% for serum lipids).

**Table 4:** Treatment according to guidelines

		**2009**		
**Medical service**		**Enrolled patients (N = 314)**	**Non-participating patients (N = 11,885)**	**P Value**
HbA1C measurement - no. (%)				<0.001
	none	40 (12.7)	4,353 (36.6)	
	1	232 (73.9)	6,867 (57.8)	
	2	42 (13.4)	653 (5.5)	
	3	0 (0.0)	15 (0.1)	
	4+	0 (0.0)	0 (0.0)	
Ophtalmoscopy - no. (%)				<0.001
	none	156 (49.7)	7,884 (66.3)	
	1+	158 (50.3)	4,004 (33.7)	
Electrocardiogram - no. (%)				<0.001
	none	155 (49.4)	7,755 (65.2)	
	1+	159 (50.6)	4,132 (34.8)	
Serum lipids - no. (%)				<0.001
	none	45 (14.3)	4,535 (38.1)	
	1+	269 (85.7)	7,353 (61.9)	
Microalbumin - no. (%)				<0.001
	none	206 (65.6)	11,288 (95.0)	
	1+	108 (34.4)	599 (5.0)	
Status of blood vessels - no. (%)				<0.001
	none	203 (64.6)	9,445 (79.4)	
	1+	111 (35.4)	2,443 (20.6)	

All variables were compared with Pearson's chi-square test.

### Costs of care and medication

Table 5 summarizes the total number and costs of outpatient services as well as medication received by both study groups as well as overall cost estimates for 2006 and 2009. As shown above, when discussing outpatient health services utilization, DMP patients received significantly fewer services in 2009 (185 vs. 191 services). However, due to the larger amount of medical as well as diagnostic services associated with a larger conformity to treatment guidelines, total costs of outpatient care were higher for patients enrolled in the DMP amounting to € 1,988 while services received by patients in regular treatment only totaled € 1,615.

**Table 5:** Costs of care and medication

		**2006**			**2009**		
		**Enrolled patients (N = 314)**	**Non-participating patients (N = 11,885)**	**P Value**	**Enrolled patients (N = 314)**	**Non-participating patients (N = 11,885)**	**P Value**
Outpatient care							
	Total no. of services recieved	138,47	135,51	<0.001	185,21	190,65	<0.001
	Total costs of service recieved	€ 1.463,37	€ 1.291,17	<0.001	€ 1.987,97	€ 1.615,06	<0.001
Medication							
	Total no. of medication recieved	50,94	50,62	0,376	63,51	62,94	0,383
	Total costs of medication recieved	€ 886,63	€ 901,77	0,195	€ 1.169,78	€ 1.206,64	0,086
	Total costs of outpatient care and medication	€ 2.350,00	€ 2.192,94	<0.001	€ 3.157,75	€ 2.821,70	<0.001
Inpatient care							
	Average days spent in hospital (all patients)	3,90	4,26	0,883	3,75	6,03	0.381
	Average costs für inpatient care*	€ 2.324,40	€ 2.538,96	n/a	€ 2.235,00	€ 3.593,88	n/a
Overall costs of care and medication		€ 4.674,40	€ 4.731,90	n/a	€ 5.392,75	€ 6.415,58	n/a

* Average costs for each day spent in hospital amounted to € 503 p.d. in 2006 and € 596 p.d. in 2009 (Embacher, 2010).

All variables were compared with the Mann–Whitney *U*-test.

Comparing these figures to average costs and utilization of outpatient care in 2006 (i.e. the year before the disease management program started), one can first observe that the average number of services as well as its associated costs have substantially risen for both groups, ranging from an increase of 25% for costs for patients receiving regular treatment to an increase of 40% of services again provided to non-participating patients. In contrast, increases in costs and outpatient services received by enrolled patients appear to be more consistent (+ 34% for services vs. + 35% for costs) resulting in a change of direction in the difference of services received from 2006 to 2009: Whereas patients who enrolled in the DMP later received significantly more services than their non-participating counterparts before the program started. The number of services provided to those patients in 2009 was significantly lower than the number for patients in regular treatment. Taking into account the increase in the differences of costs for outpatient care from 2006 to 2009 (€ 172 p.c. Vs. € 373 p.c.) these findings appear to be in line with a more elaborate pattern of outpatient care for DMP patients as presented in Table 2.

Contrary to outpatient care, DMP patients received slightly more prescribed medication (63.5 Vs. 62.9) at lower costs (€ 1,170 vs. € 1,207). The 2006 figures for number and costs of medication received show that increases in prescriptions (+25%) as well as costs (+32% for DMP patients vs. +34% for non participating patients) were quite similar for both study groups while the higher increases in costs for medication in general reflected price trends for medication in Austria.

When adding the costs for outpatient services to the medication received, the total costs of outpatient diabetes management amounted to € 3,158 for enrolled patients and € 2,822 for their non-participating counterparts in 2009. The overall difference in costs was € 336 p.c. in 2009 (p < 0.001) which was by and large caused by the reported differences in costs of outpatient care, whereas costs for medication were slightly lower for DMP patients. As for medication, 2006 figures revealed similar tendencies, though the differences in total costs were lower (€ 157 p.c.) because of differences in the patterns of outpatient care at baseline as well as in its subsequent developments.

When calculating cost estimates for inpatient care based on average days spent in hospitals for both study groups as well as average costs for each day spent in hospitals as presented in official statistics of hospital accounting [24], one can see that overall costs for care and medication were substantially lower for patients enrolled in the DMP in 2009, representing approximately 85% of the costs for patients receiving regular treatment (€ 5,393 vs. € 6,416). Comparing these figures to 2006 overall cost estimates, one can still detect higher overall costs for in- and outpatient care as well as medication for the control group (€ 4,674 vs. € 4,732), but the overall cost difference was substantially lower in 2006, reflecting only 1% of total costs.

As presented in Table 5, the statistics on inpatient care show that the increasing divergence in overall costs between the two study populations is mainly due to increasing differences in the average number of days spent in hospital and its associated costs: Whereas for patients, who enrolled in the DMP later the average number of days spent in hospital actually decreased from 2006 to 2009 (3.90 days vs. 3.75 days), the number of average days in hospital increased for non-participating patients from an average of 4.26 days in 2006 to 6.03 days in 2009. Even though overall differences in hospital days did not show statistical significance in 2009, the differences for the 2009 figures on average days spent in hospital as well as average length of stay for the subgroup of hospitalized patients (see Table 3) suggest that hospitalization rates for patients enrolled in the DMP not only differ from those of their non-participating counterparts, but also that participation in DMP leads to slightly lower utilization of inpatient care, whereas the control group experiences substantial increases.

Overall Table 5 illustrates that participation in the DMP not only led to a shift of health services utilization within outpatient care, but apparently also from inpatient to outpatient care in terms of costs, as the group of DMP patients accounted for lower costs for inpatient care and higher costs for outpatient services in 2009 as compared to corresponding figures for 2006 before the start of the diabetes DMP.

## Discussion

The results presented above show that patients enrolled in the diabetes DMP receive a more evolved pattern of outpatient services both in terms of medical services provided by specialists and ambulatory health care centers as well as in terms of specific services as put forward by medical guidelines indicating an improved process quality of outpatient care.

As far as the overall number of outpatient services received (Table 2) is concerned, these findings are generally in line with other research: Villagra & Ahmed [6] present evidence for less frequent use of outpatient services with DMP patients accounting for significantly fewer office visits and Dall et al. [7] also observed that active participation in a diabetes DMP led to a decrease in the number of ambulatory visits. Contrary to the findings of Villagra & Ahmed [6] and Dall et al. [7], who also reported lower costs for overall outpatient care, the authors identified significantly higher costs for overall outpatient care of diabetes patients receiving treatment within the DMP (Table 5), which was also found by Buntin et al. [11] when analyzing insurers data on health care use and costs for more than 12,000 diabetics.

Analyzing the mere structure of the different outpatient services as presented in Table 2, it becomes clear that increased costs for outpatient care for the DMP group are mainly due to the higher share of enrolled patients receiving specific diagnostic services according to the treatment guidelines as opposed to the group of non-participating patients and is therefore in line with the intention of disease management. Higher frequencies of these services performed ultimately lead to higher frequencies of medical specialist and diagnostic services performed, which could also be observed by Sidorov et al. [14], who reported a higher number of outpatient office visits as well as higher figures of HbA1C testing and of lipid, eye and kidney screening for DMP patients. Similarly, other studies reported evidence for a significant increase in the frequency of specific examinations such as HbA1C testing, eye exams, foot exams and cholesterol testing due to participation in the DMP [5,10].

The average number of inpatient stays as well as average length of stay (Table 3)reveals that patients enrolled in the DMP experienced fewer admissions (0.63 Vs. 0.80) and inpatient days (3.785 Vs. 6.03) per patient, reflecting a reduction in the latter of more than 40%. Again, these findings appear to be in line with the results presented by Sidorov et al. [14], Dall et al. [7] and Villagra & Ahmed et al. [6], who present evidence for a significant decline in inpatient days for DMP patients ranging from 20% to 40%. Considering the fact that the average hospitalization rates for regular diabetes patients observed in these US-based studies range between 1 and 1.5 days per patient per year [6,7,14,15], it can be shown, that even within Austria’s hospital-driven health system, DMPs lead to similar relative reductions of hospitalization rates and hence appear to be able to counterbalance the adverse governance effects primarily put forward by the characteristics of the Austrian DRG-system [23].

As observed by Villagra & Ahmed [6] and Dall et al. [7], it is the overall reduction in inpatient days that represents the most important source of savings, which is also reflected by our results on overall cost effects of DMP enrollment as presented in Table 5. While participation in the DMP leads to substantial cost advantages for inpatient care due to steady figures on average days spent in hospital as opposed to substantial increases in hospital days for non-participating diabetics from 2006 to 2009, enrollment does not tend to have an overall impact on the amount and costs of prescribed medication. Villagra and Ahmed [6] and Sidorov et al. [14] observed similar effects suggesting that disease management is likely to increase the amount of medication due to higher adherence to pharmacological regimes on the one hand, while decreasing the amount of medication due to appropriate use and better disease control on the other.

Most important, in line with the results presented in recent studies on the effects of diabetes disease management in the UK [25] and in the US [6,7], our findings indicate that even within the Austrian mandatory health insurance system, which has little competition and free patient choice in terms of outpatient care, type 2 diabetics currently enrolled in the DMP benefit from lower hospitalization rates as compared to non-participating patients. They also benefit from a different pattern of outpatient care promoting primary and specialist care associated with higher levels of medical assessments and tests according to guideline recommendations.

Yet one should acknowledge that participation in the DMP is still very low amongst type 2 diabetics covered by the Social Insurance Institution for Business and only reflects 2.5% of the target population in 2009. These low participation rates may be due to the specific characteristics of the insurer’s population, which mainly includes self-employed persons. Alternatively these figures may also reflect low overall participation rates throughout Austria, with the Social Insurance Institution of Styria leading the field with 8% of enrolled diabetics by the end of 2009 [26].

As put forward in the introduction, this study ultimately aims to evaluate the effects of diabetes disease management from an insurer’s perspective. The low DMP participation rates in Austria mean that health insurance providers seem cautious about promoting disease management. Even though our findings support the evidence for overall cost advantages for disease management, health insurance providers are very unlikely to benefit from disease management in financial terms, as more evolved outpatient services remunerated on a fee-for-service basis lead to higher costs whereas the insurance providers’ financial contributions to the centrally funded Austrian DRG-system remain constant regardless of changes in hospitalization rates. Hence, hospital operators tend to profit from disease management, as reduced hospitalization rates in terms of hospital stays cause higher remuneration for the remaining cases and consequently higher gross margins on the one hand. On the other, lower average lengths of stay may also contribute to the financial benefit of hospitals, as for the case of comparable main diagnosis groups within both study populations (secondary diagnoses do not have an impact on Austrian case groups), hospitals receive similar average lump-sum payments for enrolled and non-participating patients whereas costs associated with each particular inpatient stay vary due to the different number of days spent in hospital [22]. One should therefore keep in mind that further promotion or even adoption of diabetes disease management into the basic benefit package would have to come with the implementation of adequate remuneration schemes in order to ensure that insurance providers benefit from cost advantages.

Our study has two basic types of limitations, the first relating to methodological concerns about the impact of the assessed outcome measures on overall health outcome. Several studies report evidence that diabetes DMPs are associated with better health related quality of life [27], reduced mortality [28] and in particular intermediate outcomes associated with better control of disease such as glycated hemoglobin levels, blood pressure and cholesterol levels [5,8-10,29,30].

In our study we only had access to the insurance provider’s patient records including frequencies as well as the costs of the reimbursed health services. Information on intermediate outcomes such asHbA1C level, blood pressure, cholesterol level is not reported to health insurers (whether or not patients participated in the DMP) and can only be collected by either analyzing physicians’ own patient documentation [13] or direct inquiry of diabetes patients via questionnaires or interview [5,8]. However, as McEwen et al. [9], Stark et al. [5], Snyder et al. [19] and Rothe et al. [30] observed improved healthcare processes in terms of frequency of specific assessments performed as well as improved intermediate outcomes, it can well be assumed that the better treatment conforms to the guideline, the better overall control of diabetes.

The second type of limitations reflects methodological concerns, which are due to the design of the study as an observational retrospective cohort-study. As put forward by several authors [11-13] selection effects are likely to occur in population based DMPs. The direction of these effects is not consistent however: whereas Linder et al. [12] and Schäfer et al. [13] report that patients with a better prognosis and lower risk status as well as higher self-activity and motivation are more likely to be included in the DMP, Buntin et al. [11] found evidence for higher utilization of health services and drugs before enrolment into the DMP which may be associated with a worse health status of DMP participants as well as higher motivation or a more stable life situation.

Due to the restricted access to other relevant socio-demographic characteristics such as income, education, profession or marital status and the lack of documented diagnosis in the patient records, the authors could neither control for socio-demographic differences other than age and sex between the group of participating and non-participating patients, nor identify a specific matching control group or matched pairs as suggested by Linder et al. [12] and Miksch et al. [28] respectively.

Schäfer et al. [8] used patient documentation of primary care physicians of randomly selected diabetes patients in order to determine differences not only in social-demographic characteristics, but also in cardiovascular risk profile and patient motivation. Again, these items are not covered by patient records held by Austrian mandatory health insurance providers and physicians are only obliged to perform extended documentation for patients enrolled in the diabetes DMP [18,31].

Assessing the selection effect of our study, the specific target population of the insurance institution has to be taken into account: As pointed out in the presentation of the population studied, the Social Insurance Institution for Business only serves self-employed and retired self-employed persons as well as their close relatives (spouses, children) unless not covered by another insurance institution. As socio-economic factors such as household assets and education (along with race/ethnicity) have recently proven to be independent predictors of health decline amongst diabetics over 65 [32] and due to the fact that variances within socio-economic characteristics within the group of self-employed can be deemed to be substantially smaller than for regional subsets of the whole population (apart from the fact that particular lifestyles associated with socio-economic factors favor the acquisition of diabetes in first place) selection effects due to economic factors are unlikely to account for the majority of the observed differences between the two study groups.

In terms of data on cost and utilization of the study population before the start of the DMP on the other hand, we did find substantial differences within the costs of the services that outpatient received, indicating a higher share of more expensive services provided to patients who later enrolled themselves in the DMP. However, as pointed out above when discussing the direction of possible selection effects, it remains unclear whether these differences at baseline are due to the higher patient involvement for the DMP group or a worse status of health within this group resulting in more expensive services. Considering this potential selection effect due to differences in individual as well as disease-related characteristics of diabetes patients, the comparison of the 2006 statistics with their corresponding figures for 2009 provides evidence of the efficiency of the Austrian diabetes DMP: Patients who decided to participate in the program experienced a reduction in overall days spent in hospital as well as lower increases in the number of outpatient services. This resulted in overall lower costs for care and medication as compared to their counterparts receiving regular diabetes treatment.

## Conclusions

The overall aim of the study was to assess whether participation in the Austrian diabetes DMP ultimately leads to differences in the use of health services and its subsequent costs as well as treatment according to guidelines in order to control for patient safety and process quality of care. This question is of particular relevance for health insurance providers in order to determine whether further promotion of DMPs with voluntary participation should be pursued. As insurance providers adopt a population based perspective, the analysis of the pattern of health care utilization must be performed by comparing the actual group of enrolled patients to the actual group of non-participating diabetics in order to estimate real cost effects.

As such, the Austrian diabetes DMP appears to be effective in promoting a more evolved pattern of outpatient services delivered and apparently less hospitalization rates, even though the program’s current rather loose regime of voluntary participation and modest financial incentives for physicians. Notwithstanding methodological concerns about selection biases, one should remember that analysis and evaluation of particular health policies such as disease management is always context specific and hence needs thorough analysis of human behavior in order to gain insights into the mechanisms which can then promote policy learning [33]. In fact, the authors analyzed the Austrian diabetes DMP with emphasis on its internal validity as a whole. Whether the shift in outpatient care towards services in line with treatment guidelines was caused by physician promotion or changed patient demand as a consequence of education or both could not be differentiated. Moreover, more elaborated approaches to diabetes management such as the chronic care model in integrated care settings which may even lead to better results [30,34] were beyond the scope of the study, too, as the authors could only analyze the effects of the established program within the historic cohort group

As in other health systems, health insurance providers and public health politicians face the challenge of whether or not to adopt disease management as a standard benefit [4] and of deciding how particular programs can be set up in order to gain maximum benefit. Our research led to promising results in favor of diabetes disease management. However, due to the small number of patients enrolled in the program, future inclusion of more patients into disease management – either via increased promotion or via integration into the standard benefit package – should be accompanied by constant monitoring. In line with other research [4,11-15,29] we suggest setting up randomized controlled trials on regional scales in order to enhance empirical evidence on the effects of the Austrian diabetes DMP with particular regard to its intermediate outcomes.

## Competing Interests

The evaluation study was mandated to the Division for Health Policy, Administration and Law at UMIT by the Social Insurance Institution for Business. Michael Mueller holds the position of the head of the Unit for Health Management at the Social Insurance Institution for Business.

Even though the authors did not encounter any attempt of influence during the course of the research project (HO, VH, MM) as well as professional activity (MM), potential conflicts of interest exist due to the contractual relationships with the Social Insurance Institution for Business.

## Authors’ contributions

HO and MM designed the study. MM supervised the data collection, HO analyzed the data and HO and MM interpreted the findings. HO drafted the manuscript, MM and VH reviewed the different phases of the manuscript. All authors read and approved the final manuscript.

## Pre-publication history

The pre-publication history for this paper can be accessed here:

http://www.biomedcentral.com/1471-2458/12/490/prepub
